# Cysteine conjugate beta-lyase 2 (*CCBL2*) expression as a prognostic marker of survival in breast cancer patients

**DOI:** 10.1371/journal.pone.0269998

**Published:** 2022-06-30

**Authors:** Xiangyu Meng, Ling Wang, Miao He, Zhaoying Yang, Yan Jiao, Yubo Hu, Keren Wang

**Affiliations:** 1 Department of Breast Surgery, China-Japan Union Hospital of Jilin University, Changchun, Jilin, China; 2 Department of Obstetrics and Gynecology, The Second Hospital of Jilin University, Changchun, Jilin, China; 3 Department of Anesthesia, The Second Hospital of Jilin University, Changchun, Jilin, China; 4 Department of Hepatobiliary and Pancreatic Surgery, The First Hospital of Jilin University, Changchun, Jilin, China; 5 Department of Anesthesia, China-Japan Union Hospital of Jilin University, Changchun, Jilin, China; University of Salerno, ITALY

## Abstract

**Objective:**

Cysteine conjugate beta-lyase 2 (*CCBL2*), also known as kynurenine aminotransferase 3 (*KAT3*) or glutamine transaminase L (*GTL*), plays an essential role in transamination and cytochrome P450. Its correlation with some other cancers has been explored, but breast cancer (BC) not yet.

**Methods:**

The mRNA and protein expression of *CCBL2* in BC cell lines and patient samples were detected by RT-qPCR and immunohistochemistry (IHC). BC patients’ clinical information and RNA-Seq expression were acquired via The Cancer Genome Atlas (TCGA) database. Patients were categorized into high/low *CCBL2* expression groups based on the optimal cutoff value (8.973) determined by receiver operating characteristic (ROC) curve. We investigated *CCBL2* and clinicopathological characteristics’ relationship using Chi-square tests, estimated diagnostic capacity using ROC curves and drew survival curves using Kaplan–Meier estimate. We compared survival differences using Cox regression and externally validated using Gene Expression Omnibus (GEO) database. We evaluated enriched signaling pathways using gene set enrichment analysis (GSEA), explored CCBL2 and relevant genes’ relationship using tumor immunoassay resource (TIMER) databases and used the human protein atlas (HPA) for pan-cancer analysis and IHC.

**Results:**

*CCBL2* was overexpressed in normal human cell lines and tissues. *CCBL2* expression was lower in BC tissues (n = 1104) than in normal tissues (n = 114), validated by GEO database. Several clinicopathologic features were related to *CCBL2*, especially estrogen receptor (ER), progesterone receptor (PR) and clinical stages. The low expression group exhibited poor survival. *CCBL2*’s area under curve (AUC) analysis showed finite diagnostic capacity. Multivariate cox-regression analysis indicated *CCBL2* independently predicted BC survival. GSEA showed enriched pathways: early estrogen response, MYC and so on. *CCBL2* positively correlated with estrogen, progesterone and androgen receptors. *CCBL2* was downregulated in most cancers and was associated with their survival, including renal and ovarian cancers.

**Conclusions:**

Low *CCBL2* expression is a promising poor BC survival independent prognostic marker.

## Introduction

Breast cancer (BC) is the most diagnosed cancer in women, accounting for 11.7% of all types of cancers worldwide and the highest morbidity rate in women [1–3]. This commonly diagnosed malignant tumor is also the leading cause of cancer deaths worldwide, just after lung cancer [4]. Approximate 2.1 million people were diagnosed with BC in 2018 [5]. As a heterogeneous disease, various biomarker-based diagnostic and prognostic approaches have emerged in recent years. ER, PR and human epidermal growth factor receptor-2 (HER2) have served as both diagnostic and prognostic biomarkers of BC [6]. Nowadays, with advances in sequencing technology, DNA methylation, miRNAs, autoantibodies, lipidomics and proteomics as well as identification of multiparameter gene signatures have facilitated the early diagnosis and prognosis of breast carcinoma [7–10]. These latest studies have sparked our interest in mining genes as biomarkers associated with BC.

*CCBL2* has been found in mouse, rat, and human whose mRNA is widely expressed in several organs such as the liver, kidney, heart, and neuroendocrine tissues. However, the highest expression of *CCBL2* is found in the kidney [11, 12]. *CCBL2* can effectively catalyze the transamination of glutamine, methionine, histidine, phenylalanine, cysteine, asparagine, and kynurenine (KYN) to kynurenic acid (KYNA) as well as the pathway of drug metabolism by cytochrome P450 [11, 13]. All of these functions are involved in the important processes in human amino acid metabolism. According to the HUGO Gene Nomenclature Committee (HGNC), *CCBL2* is identical to kynurenine aminotransferase 3 (*KAT3*) and glutamine transaminase L (*GTL*) genes [12, 14]. In mammalian cells, the essential amino acid tryptophan is degraded mainly through the kynurenine pathway. Kynurenine aminotransferases (*KATs*) catalyze the synthesis of KYNA, which is a metabolite of tryptophan and an endogenous antagonist of N-methyl-D-aspartate and alpha 7-nicotinic acetylcholine receptors [15–17]. And KYNA is a recognized neuroprotective and anticonvulsant agent involved in synaptic transmission and in the pathophysiology of various neurological disorders(11). Abnormal expression levels of *CCBL2* are involved in the pathophysiological process of kidney injury, hospital-acquired VTE, depression and neurological disorders [12, 15, 18–22]. Recently, *GTK* (Glutamine Transaminase K, which is identical to *KAT1* and *CCBL1*) has been reported to play an important role in pancreatic tumorigenesis through the glutamine pathway and cysteine conjugate beta-lyase (*CCBL*) had close relation with kidney cancer. Glutamine, as one of the catalytic substrates of *CCBL2*, plays biosynthetic roles in cells, as it is used in the biosynthesis of amino acids, proteins, lipids, and nucleotides which are essential to cell division, especially in cancer cells, also known as glutamine addiction, reported to be concerned with the process of pancreatic cancer. Furthermore, cysteine conjugate beta-lyase (*CCBL*) was found to be closely associated with the development of kidney cancer. Studies have shown that variants in the *CCBL2* gene were significantly associated with the risk of chronic kidney disease due to a defect in reductive metabolism that leads to the formation of a cysteine conjugate, which is then converted to an active metabolite [20, 22, 23]. The findings piqued our curiosity in the role of the *CCBL* family member, *CCBL2*, in breast tissues, as well as the unknown association between *CCBL2* and BC, which is now the most frequently diagnosed cancer worldwide. Moreover, we assessed whether *CCBL2* could serve as a prognostic marker of survival in patients with BC.

Therefore, to initially ascertain whether *CCBL2* expression levels affect BC prognosis, we studied the correlation between *CCBL2* expression in BC tissues and clinicopathological characteristics, as well as with the survival status of patients with BC through analysis of The Cancer Genome Atlas-Breast Invasive Carcinoma (TCGA-BRCA) level 3 data. Additionally, the results were validated using Gene Expression Omnibus (GEO) datasets. At both mRNA and protein levels, the expression of *CCBL2* was verified with real-time quantitative polymerase chain reaction (RT-qPCR) and immunohistochemistry (IHC) staining in human protein atlas (HPA) dataset, respectively. Furthermore, a pan-cancer analysis of *CCBL2* was performed to explore the correlation between *CCBL2* and various cancers.

## Methods

### Breast cell lines

During this study, two types of human BC cells, MCF-7 and MDA-MB-231, and human normal breast epithelial cell lines MCF-10A were used. MCF-7 cell lines, regarded as type of luminal, were cultured in DMEM (Gibco, USA). MDA-MB -231, regarded as type of basal, were grown in RPMI 1640 (Gibco, USA). Both cells were supplemented with 10% fetal calf serum (Gibco, USA) and 1% Penicillin-Streptomycin Solution (Beyotime China). MCF-10A cell lines were cultured in DMEM-F12 (Gibco, USA) with 5% equine serum, 1% Penicillin-Streptomycin Solution, 20ng/ml epidermal growth factor, 0.5ug/ml hydrocortisone, 0.1ug/ml cholera toxin and 10ug/ml insulin. The cell lines mentioned above were all obtained from the American Type Culture Collection (ATCC, USA) and cultured in a humid atmosphere of 5% CO_2_ at 37°C.

### Real-Time Quantitative Polymerase Chain Reaction (RT-qPCR)

The total RNA was isolated using the TRIzol reagent (Invitrogen, USA) and reverse transcription was implemented using the HiScript Ⅲ RT SuperMix for qPCR with gDNA wiper (Vazyme Biotech) to synthesize cDNA following the manufacturer’s instructions. The RT-qPCR was performed by ChamQ Universal SYBR qPCR Master Mix (Vazyme Biotech) and run by the Mastercycler Ep Realplex (Eppendorf, Hamburg, Germany). The relative gene expression fold change was normalized using beta-tubulin 2A (TUBB2A) as an internal control and compared with MCF-10A. The primers sequences used in this study were as follows: *CCBL2*, F: 5ʹ-ATC CTT GTG ACA GTA GGA GCA-3ʹ, R: 5ʹ-GGG CTC ATA GCA GTC ATA GAA AG-3ʹ; TUBB2A, F: 5ʹ-TTG GGA GGT CAT CAG CGA TGA G-3ʹ, R: 5ʹ-AGG CTC CAG ATC CAC CAG GAT G-3ʹ. The independent experiments were performed at least three times.

### Gene Set Enrichment Analyze (GSEA)

GSEA software3.0. (http://software.broadinstitute.org/gsea/downloads.jsp) was applied for Gene Set Enrichment Analysis (GSEA). The normalized enrichment score (NES) was obtained by alignment analysis 1000 times with FDR (false discovery rates)<0.25 and NOM p<0.05.

### Gene correlation analysis

Tumor Immunoassay Resource (TIMER) databases (https://cistrome.shinyapps.io/timer/) (data based on TGGA) were employed to explore the association between *CCBL2* and estrogen receptor 1 (*ESR1*), estrogen receptor 2 (*ESR2*), progesterone receptor (*PGR*), androgen receptor (*AR*), cytochrome P450 2B6 (*CYP2B6*), ribosomal protein S6 kinase B1(*RPS6KB1*) and *MYC* genes. Correlation analysis drew the expression scatterplots between a pair of genes in breast cancer, together with the Spearman’s rho value and estimated statistical significance.

### Pan-cancer analysis of *CCBL2*

The expression differences between normal and tumor tissues were analyzed by TIMER databases (data based on TCGA). Analysis of survival probability of *CCBL2* in pan-cancer was completed systemically in HPA databases (https://www.proteinatlas.org/). Relevant immunohistochemistry (IHC) staining images were also obtained from HPA.

### Data mining

Breast cancer patients’ data of the clinical information as well as the level 3 RNA-Seq expression were acquired via TCGA database (https://cancergenome.nih.gov/), involving normal tissues (n = 114) and BC tissues (n = 1104). The analysis process applied the RNA-Seq by Expectation Maximization (RSEM) expression values. GEO datasets (GSE42568, GSE71053) [24, 25] were obtained via the GEO database (https://www.ncbi.nlm.nih.gov/geo/).

### Statistical analysis

Using the ggplot2 package in R, the differential expression of discrete variables was processed into visible boxplots with Wilcoxon and Kruskal-Wallis test. Based on the optimal cutoff value (8.973) determined by the ROC curve, patients were categorized into high and low *CCBL2* expression groups. With the implement of Chi-square test as well as Fisher exact test in R program (version 3.5.2), the analysis of relationship between the expression of *CCBL2* and clinicopathological characteristics was performed. ROC curves of the subjects were plotted to estimate their diagnostic ability by applying the ROC package. Adopting the survival package of R, we used the Kaplan-Meier curves to compare respective differences in overall survival (OS) and relapse-free survival (RFS) between high and low groups. Kaplan-Meier Plotter (https://kmplot.com/analysis/) was used to further explore the relationship between the prognosis of patients with endocrine therapy and *CCBL2* expression. Log-rank test was used for p values calculation. The clinicopathological characteristics were selected by the univariate and multivariate cox regression analysis. Independent experiment of RT-qPCR was done three times and measured data expressed as mean ± standard deviation. The result of RT-qPCR was plotted by GraphPad Prism 8. P value<0.05 was the significance threshold.

## Results

### *CCBL2* expression in BC cell lines and tissues

**Fig 1:**
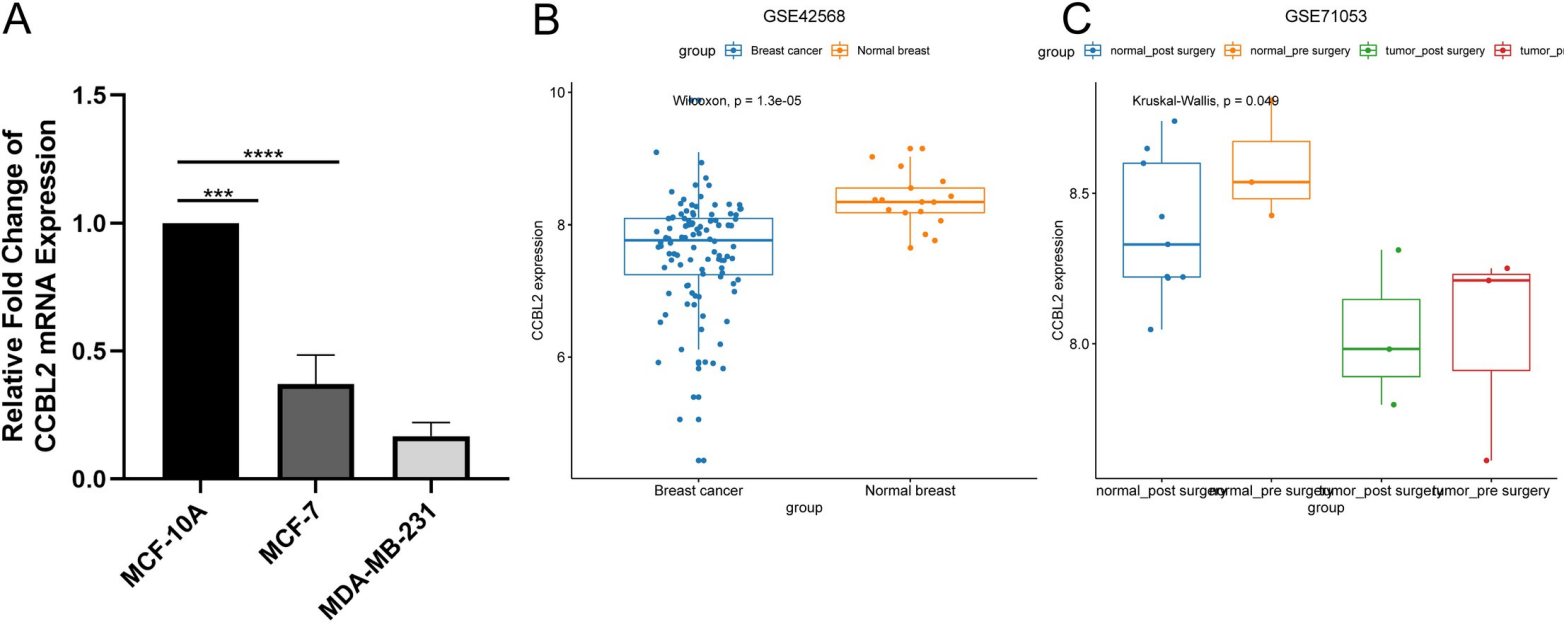
Overexpressed *CCBL2* in normal breast. MCF-7 and MDA-MB-231 mRNA expression was detected lower than MCF-10A and MCF-7 was higher than MDA-MB-231 (A) (*p<0.05, **p<0.01). Microarrays related to *CCBL2* expression in normal breast and breast cancer were shown (B and C).

**Fig 2:**
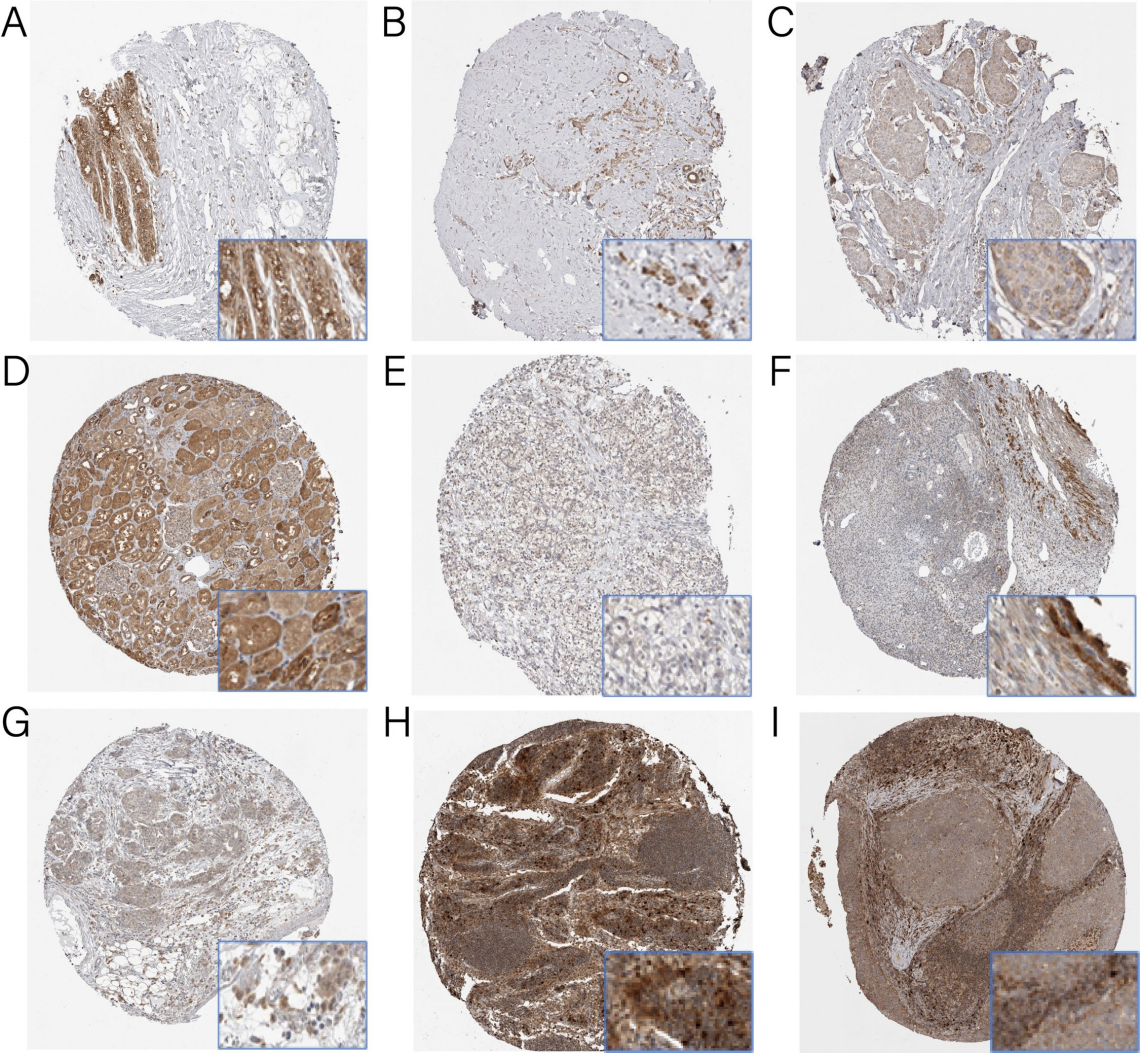
Immunohistochemistry staining for *CCBL2*. The expression of *CCBL2* in breast cancer (B and C), renal cancer (E), ovarian cancer (G) and head and neck cancer (I) cells were decreased than in respective normal tissues (A, D, F and H). In breast cancer, infiltrating lobular carcinoma (B) expressed more *CCBL2* than infiltrating ductal carcinoma (C).

*CCBL2* was overexpressed in the human normal breast epithelial cell line MCF-10A while downregulated in BC cell lines (Fig 1A) and tissues (Fig 2A–2C). In particular, MCF-7 cells showed higher *CCBL2* expression than MDA-MB-231 cells. Out of a total of 24 samples of BC, 22 of them exhibited moderate IHC staining for *CCBL2*, other two of them showed weak IHC staining. However, normal tissues exhibited strong IHC staining for *CCBL2* (Fig 2A–2C). This result indicated that *CCBL2* expression varied at the protein level. In addition, it was validated in microarrays GSE42568 (p = 1.3e-05) and GSE71053 (p = 0.0490) that *CCBL2* expression of BC was lower in tumor tissue than in normal breast tissues (Fig 1B and 1C).

### Patient characteristics

Based on the TCGA-BRCA level 3 data, Table 1 showed the clinical characteristics of tumor samples, such as molecular subtype, histological type, menopause status, radiation therapy, margin status, neoadjuvant treatment, targeted molecular therapy, ER, PR, HER-2, TNM stage, clinical stage, vital status, lymph node status and sample type.

**Table 1:** Clinical characteristics of TCGA-BRCA level 3 cohort.

Characteristics	Numbers of cases (%)
***CCBL2***	
High	746(67.57)
Low	358(32.43)
**Age**	
<60	589(53.45)
> = 60	513(46.55)
**Gender**	
Female	1090(98.73)
Male	12(1.09)
NA	2(0.18)
**Histological type**	
Infiltrating Ductal Carcinoma	790(71.56)
Infiltrating Lobular Carcinoma	204(18.48)
Other	107(9.69)
NA	3(0.27)
**Molecular subtype**	
Basal	142(12.86)
HER-2	67(6.07)
Lum A	422(38.22)
Lum B	194(17.57)
Normal	24(2.17)
NA	255(23.1)
**ER**	
Indeterminate	2(0.18)
Negative	239(21.65)
Positive	813(73.64)
NA	50(4.53)
**PR**	
Indeterminate	4(0.36)
Negative	345(31.25)
Positive	704(63.77)
NA	51(4.62)
**HER-2**	
Equivocal	180(16.3)
Indeterminate	12(1.09)
Negative	565(51.18)
Positive	164(14.86)
NA	183(16.58)
**T classification**	
T1	281(25.45)
T2	640(57.97)
T3	138(12.5)
T4	40(3.62)
TX	3(0.27)
NA	2(0.18)
**N classification**	
N0	516(46.74)
N1	367(33.24)
N2	120(10.87)
N3	79(7.16)
NX	20(1.81)
NA	2(0.18)
**M classification**	
M0	917(83.06)
M1	22(1.99)
MX	163(14.76)
NA	2(0.18)
**Stage**	
I	182(16.49)
II	626(56.7)
III	252(22.83)
IV	20(1.81)
X	14(1.27)
NA	10(0.91)
**Lymph node status**	
No	28(2.54)
Yes	697(63.13)
NA	379(34.33)
**Vital status**	
Deceased	155(14.04)
Living	947(85.78)
NA	2(0.18)
**Sample type**	
Metastatic	7(0.63)
Primary tumor	1097(99.37)
**Menopause status**	
Inde	34(3.08)
Peri	40(3.62)
Post	706(63.95)
Pre	231(20.92)
NA	93(8.42)
**Margin status**	
Close	31(2.81)
Negative	922(83.51)
Positive	79(7.16)
NA	72(6.52)
**Radiation therapy**	
NO	445(40.31)
YES	557(50.45)
NA	102(9.24)
**Neoadjuvant treatment**	
No	1088(98.55)
Yes	13(1.18)
NA	3(0.27)
**Targeted molecular therapy**	
NO	46(4.17)
YES	533(48.28)
NA	525(47.55)
**Os**	
Alive	933(85.83)
Dead	154(14.17)
**Rfs**	
Relapse-free	816(89.47)
Relapse	96(10.53)

Abbreviation: ER: estrogen receptor; PR: progesterone receptor; HER-2: human epidermal growth factor-2; T: tumor; M: metastasis; N: node; OS: overall survival; RFS: relapse-free survival; NA: not available.

### *CCBL2* expression in BC

**Fig 3:**
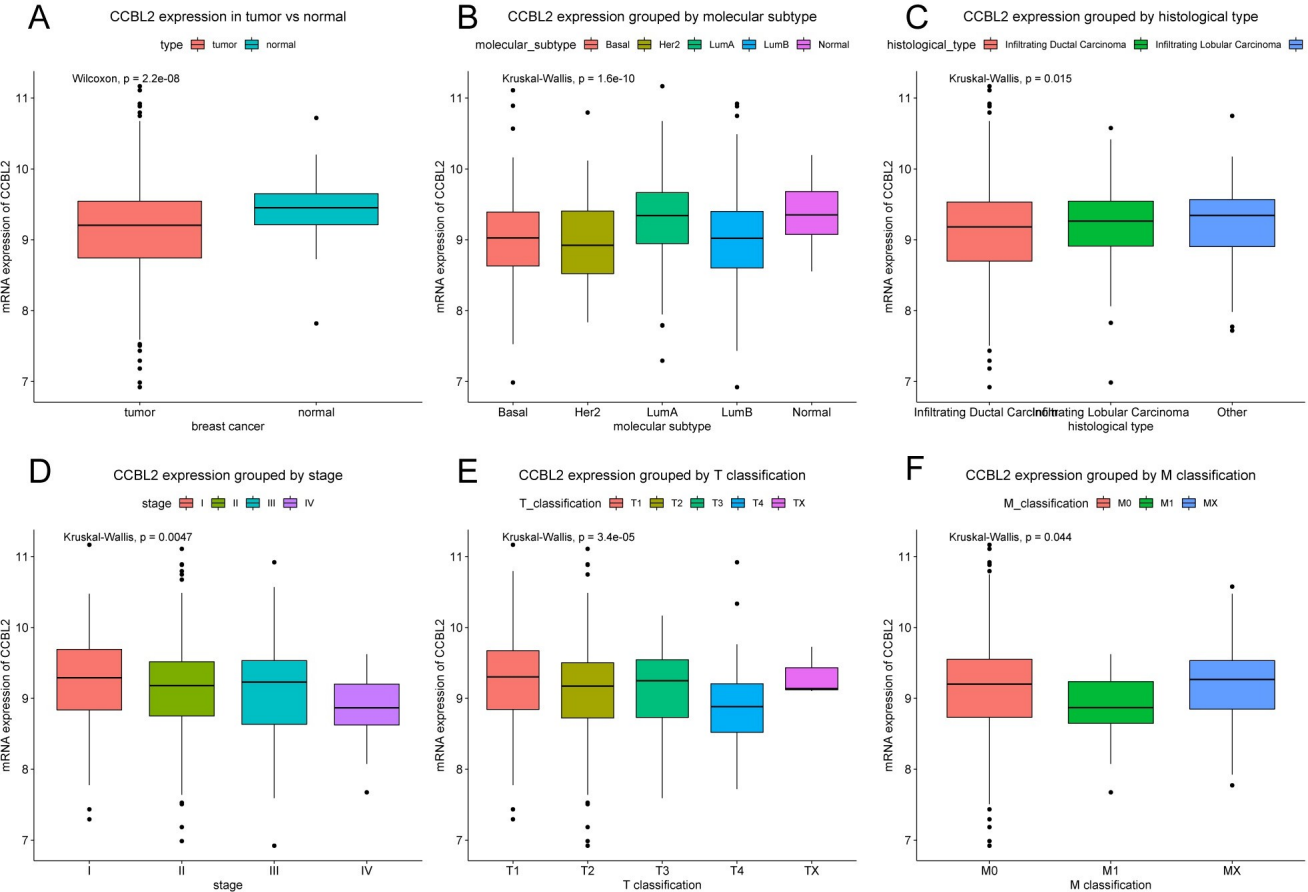
Differences in *CCBL2* expression shown in boxplots. The subgroups included type (A), molecular subtype (B), histological type (C), clinical stage (D), T classification (E), and M classification (F). (p < 0.05).

Compared with that in normal tissues (n = 114), the expression of *CCBL2* was lower in BC tissues (n = 1104; p = 2.2e-08). Additionally, drawn in boxplots, *CCBL2* expression varied with the molecular subtype (p = 1.6e-10), histological type (p = 0.0150), clinical stage (p = 0.0047), T classification (p = 3.4e-05), M classification (p = 0.0440) (Fig 3), while no statistical difference in patient age, menopause status, sample type, N classification, sample type etc. (data not shown).

### The correlation between clinicopathological characteristics and *CCBL2* expression in BC

Based on the optimal cutoff value (8.973) determined by the ROC curve, patients were categorized into high and low *CCBL2* expression groups. Results of Chi-square or Fisher exact test demonstrated that several clinicopathologic features, including histological type (p = 0.0010), molecular subtype (p = 0.0005), ER (p = 0.0005), PR (p = 0.0005), HER2 (p = 0.0085), T classification (p = 0.0130), M classification (p = 0.0210), vital status (p = 0.0025), stage (p = 0.0320), OS (p = 0.0025) and RFS (p = 0.0375), were correlated with *CCBL2* expression (Table 2).

**Table 2:** Correlations of *CCBL2* expression in BC tissues with clinicopathologic features.

Clinical characteristics	Variable	No. of cases	*CCBL2* expression		χ2	P value
			High n (%)	Low n (%)		
Age	<60	589	404 (68.59)	185 (31.41)	0.7858	0.4108
	> = 60	513	339 (66.08)	174 (33.92)		
Gender	Female	1090	736 (67.52)	354 (32.48)	0.4564	0.5437
	Male	12	7 (58.33)	5 (41.67)		
Histological type	Infiltrating Ductal Carcinoma	790	505 (63.92)	285 (36.08)	16.1545	**0.001**
	Infiltrating Lobular Carcinoma	204	156 (76.47)	48 (23.53)		
	Other	107	82 (76.64)	25 (23.36)		
Molecular subtype	Basal	142	82 (57.75)	60 (42.25)	49.4216	**0.0005**
	Her2	67	35 (52.24)	32 (47.76)		
	LumA	422	332 (78.67)	90 (21.33)		
	LumB	194	110 (56.70)	84 (43.30)		
	Normal	24	19 (79.17)	5 (20.83)		
ER	Indeterminate	2	1 (50.00)	1 (50.00)	19.3441	**0.0005**
	Negative	239	134 (56.07)	105 (43.93)		
	Positive	813	578 (71.09)	235 (28.91)		
PR	Indeterminate	4	1 (25.00)	3 (75.00)	20.1547	**0.0005**
	Negative	345	205 (59.42)	140 (40.58)		
	Positive	704	507 (72.02)	197 (27.98)		
HER2	Equivocal	180	129 (71.67)	51 (28.33)	11.7483	**0.0085**
	Indeterminate	12	9 (75.00)	3 (25.00)		
	Negative	565	382 (67.61)	183 (32.39)		
	Positive	164	91 (55.49)	73 (44.51)		
T classification	T1	281	207 (73.67)	74 (26.33)	12.7439	**0.013** ^**b**^
	T2	640	422 (65.94)	218 (34.06)		
	T3	138	91 (65.94)	47 (34.06)		
	T4	40	20 (50.00)	20 (50.00)		
	TX	3	3 (100)	0 (0)		
N classification	N0	516	350 (67.83)	166 (32.17)	3.4757	0.5072
	N1	367	255 (69.48)	112 (30.52)		
	N2	120	73 (60.83)	47 (39.17)		
	N3	79	51 (64.56)	28 (35.44)		
	NX	20	14 (70.00)	6 (30.00)		
M classification	M0	917	613 (66.85)	304 (33.15)	7.8213	**0.021**
	M1	22	10 (45.45)	12 (54.55)		
	MX	163	120 (73.62)	43 (26.38)		
Stage	I	182	134 (73.63)	48 (26.37)	10.766	**0.032**
	II	626	421 (67.25)	205 (32.75)		
	III	252	164 (65.08)	88 (34.92)		
	IV	20	8 (40.00)	12 (60.00)		
	X	14	10 (71.43)	4 (28.57)		
Lymph node status	No	28	18 (64.29)	10 (35.71)	0.5126	0.5277
	Yes	697	492 (70.59)	205 (29.41)		
Vital status	Deceased	155	88 (56.77)	67 (43.23)	9.3118	**0.0025**
	Living	947	655 (69.17)	292 (30.83)		
Sample type	Metastatic	7	4 (57.14)	3 (42.86)	0.3367	0.6982
	Primary Tumor	1097	740 (67.46)	357 (32.54)		
Menopause status	Inde	34	24 (70.59)	10 (29.41)	3.1873	0.3683
	Peri	40	29 (72.50)	11 (27.50)		
	Post	706	462 (65.44)	244 (34.56)		
	Pre	231	164 (71.00)	67 (29.00)		
Margin status	Close	31	23 (74.19)	8 (25.81)	0.9229	0.6322
	Negative	922	617 (66.92)	305 (33.08)		
	Positive	79	55 (69.62)	24 (30.38)		
Radiation therapy	NO	445	290 (65.17)	155 (34.83)	1.9235	0.1859
	YES	557	386 (69.30)	171 (30.70)		
Neoadjuvant treatment	NO	1088	735 (67.56)	353 (32.44)	0.2119	0.7686
	YES	13	8 (61.54)	5 (38.46)		
Targeted molecular therapy	NO	46	32 (69.57)	14 (30.43)	0.0035	1
	YES	533	373 (69.98)	160 (30.02)		
OS	Alive	933	646 (69.24)	287 (30.76)	9.7778	**0.0025**
	Dead	154	87 (56.49)	67 (43.51)		
RFS	Relapse-free	816	566 (69.36)	250 (30.64)	4.8181	**0.0375**
	Relapse	96	56 (58.33)	40 (41.67)		

Abbreviations: Bold values of P < 0.05 indicate statistically significant correlations.

^b^Fisher’s exact test.

Note: High n (%) and low n (%) added up to 100% in each subgroup. For example, high BBCL2 expression n (%) of “Age<60” = 404/589 = 68.59%; low CCBL2 expression n (%) of “Age<60” = 185/589 = 31.41%

### Diagnostic capacity of *CCBL2* expression

**Fig 4:**
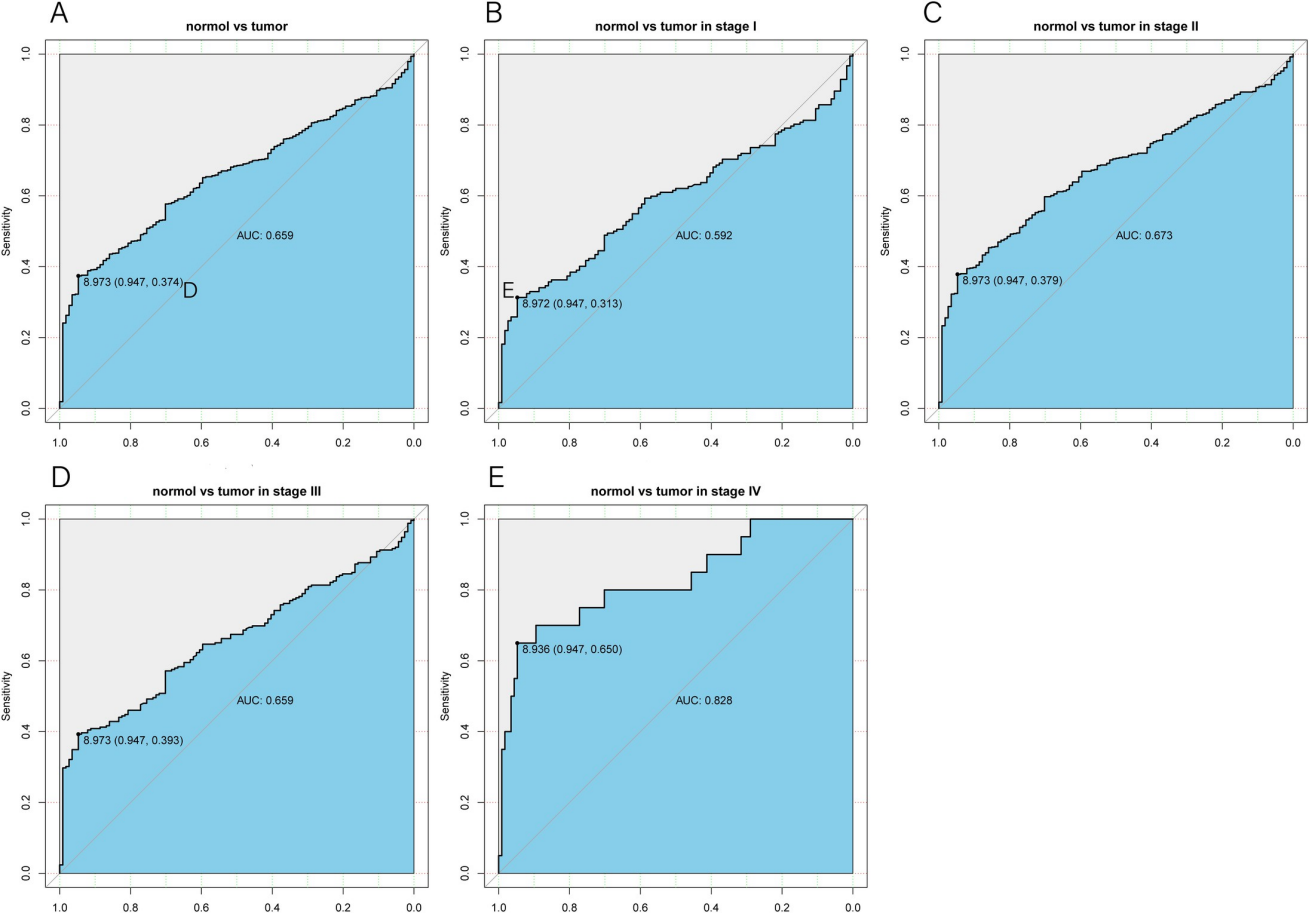
The ROC curve of *CCBL2* in breast carcinoma cohort. Normal and tumor samples (A). The AUC (0.659) indicated a limited diagnostic capability. Subgroup analyses: Stage Ⅰ (B) (AUC: 0.592), Stage Ⅱ (C) (AUG:0.673), Stage Ⅲ (D) (AUG:0.659), Stage Ⅳ (E) (AUG:0.828). Abbreviations: AUC, area under the curve; ROC, receiver operating characteristic.

The ROC curve was plotted to assess the diagnostic capacity of *CCBL2* and the area under the curve (AUC) showed a value of 0.659, implying a finite diagnostic capacity. In the subgroup analysis of different stages, *CCBL2* showed a relatively valuable diagnostic capacity in patients with stage IV BC (AUG: 0.828) (AUC values of BC stages I, II, and III were 0.592, 0.673, and 0.659 respectively) (Fig 4).

### Correlation between *CCBL2* expression and survival of patients with BC

**Fig 5:**
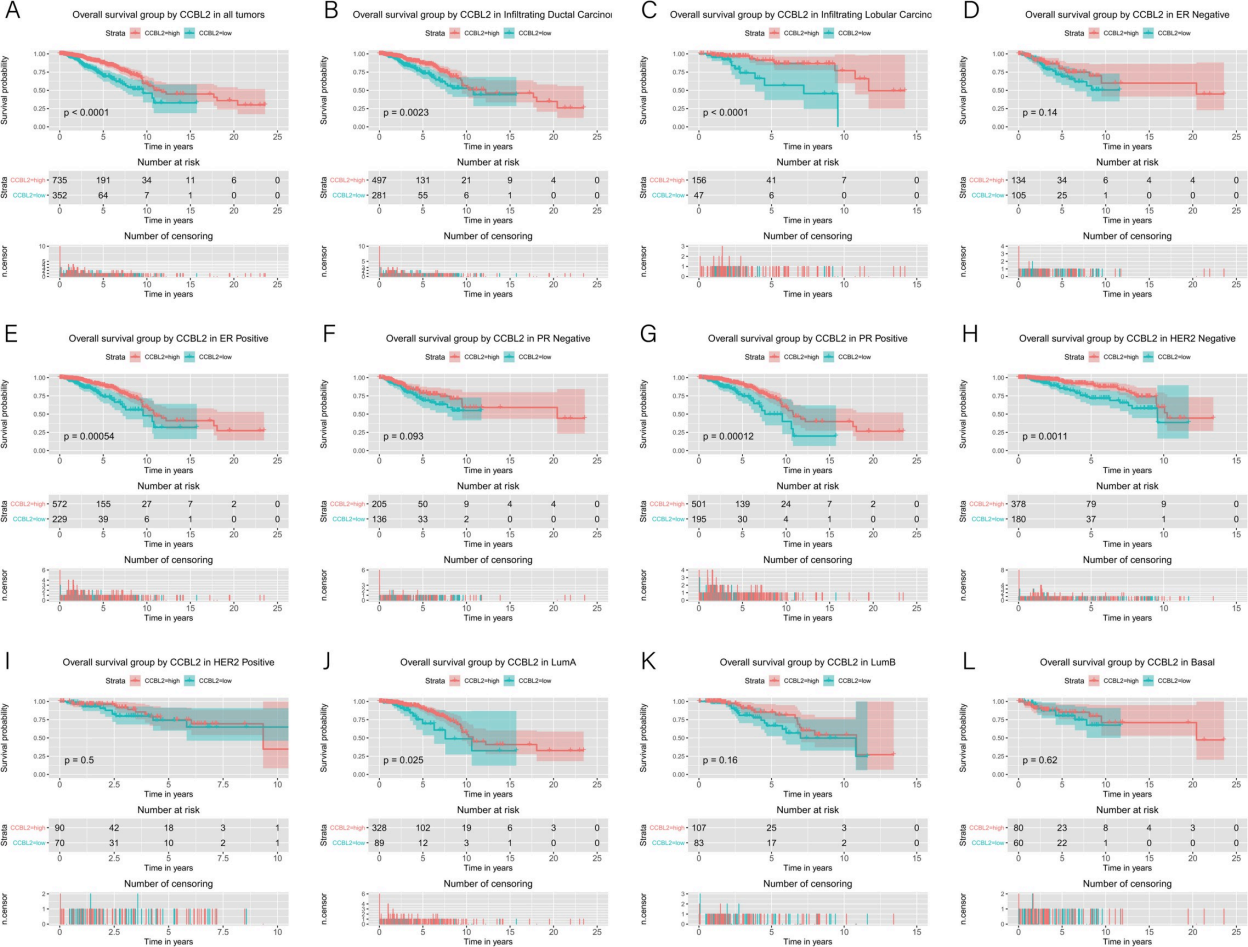
Kaplan–Meier curves of overall survival in breast cancer according to *CCBL2* expression in breast cancer tissues. Overall survival analysis and subgroup analyses of histological type (B and C), ER (D and E), PR (F and G), HER-2 (H and I) and molecular subtype (J, K and L). High *CCBL2* expression had relations with the high overall survival. (p < 0.0001) (A).

**Fig 6:**
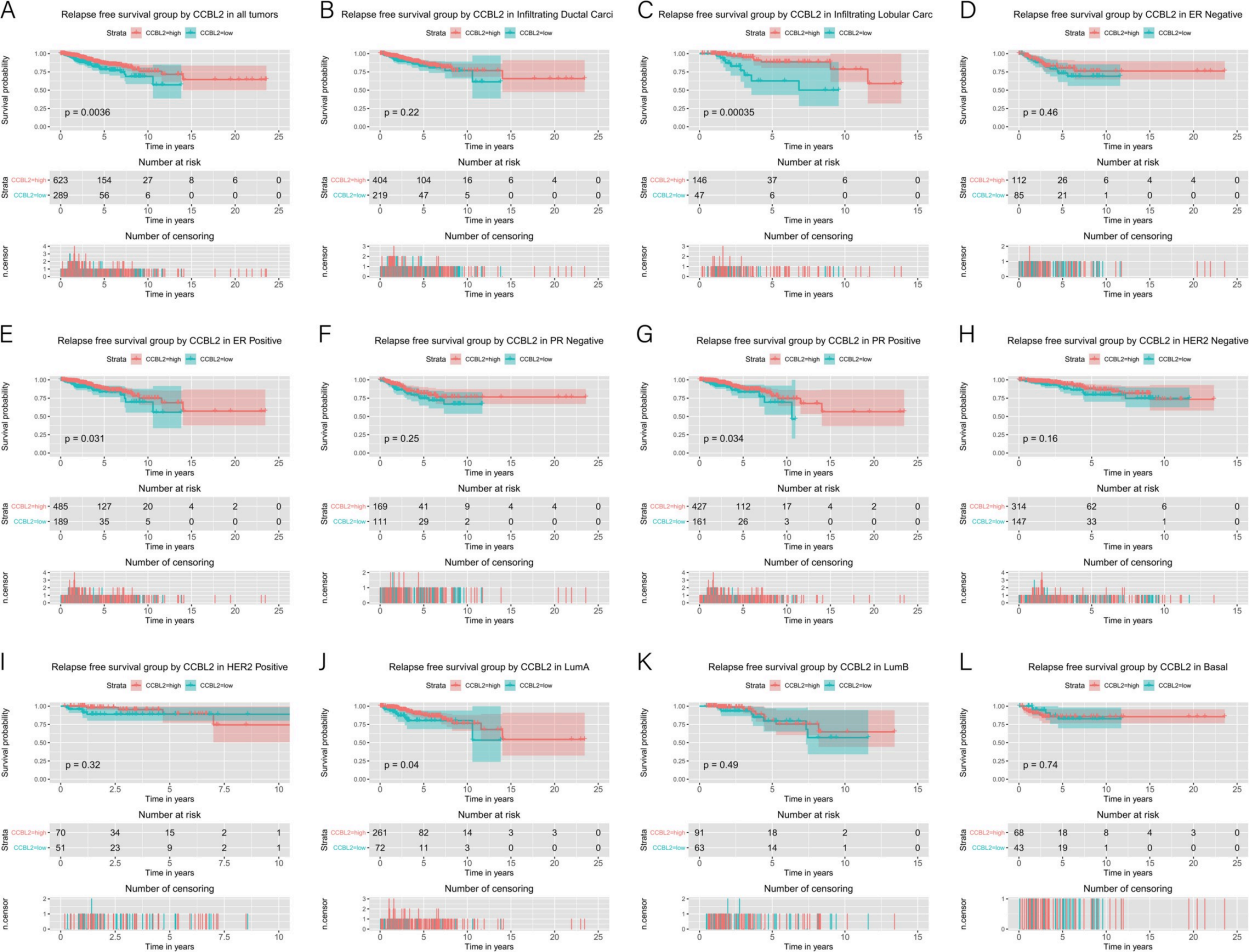
Kaplan–Meier curves of relapse free survival in breast cancer according to *CCBL2* expression in breast cancer tissues. Relapse free survival analysis and subgroup analyses of histological type (B and C), ER (D and E), PR (F and G), HER-2 (H and I) and molecular subtype (J, K and L). High *CCBL2* expression had relations with the high relapse free survival (p = 0.0036) (A).

**Fig 7:**
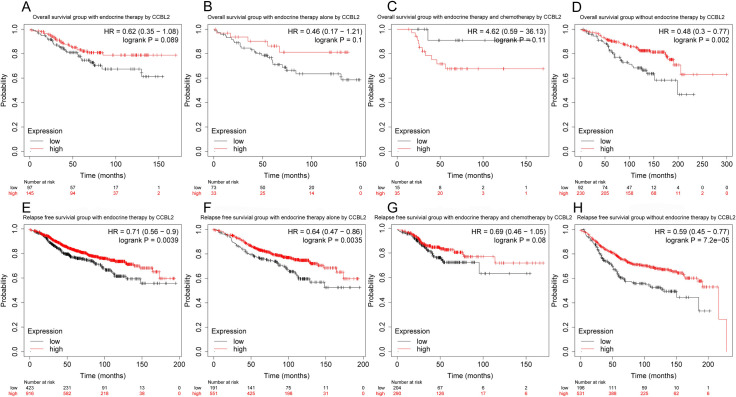
Kaplan–Meier curves of overall survival and relapse free survival in ER-positive breast cancer according to *CCBL2* expression in breast cancer tissues. Subgroups analysis included the overall survival and relapse free survival of patients with endocrine therapy (A and E), with endocrine therapy alone (B and F), with endocrine therapy and chemotherapy (C and G), and without endocrine therapy (D and H). P value<0.05 was the significance threshold.

The correlation between *CCBL2* expression and survival of patients with BC was determined using Kaplan–Meier curves. The log-rank tests indicated that low *CCBL2* expression was associated with a low overall survival (OS) rate (p<0.0001) (Fig 5) as well as a low relapse-free survival (RFS) rate (p = 0.0036) (Fig 6). Subgroup analysis revealed that low CCBL2 expression was correlated with low OS in patients with ER-positive BC (p = 0.0005), PR-positive BC (p = 0.0001), HER-2-negative BC (p = 0.0011), infiltrating ductal carcinoma (p = 0.0023), infiltrating lobular carcinoma (p<0.0001), and luminal A (p = 0.0250) (Fig 5). The analysis also revealed that low CCBL2 expression was associated with low RFS in patients with ER-positive BC (p = 0.0310), PR-positive BC (p = 0.0340), luminal A BC (p = 0.0400), and infiltrating ductal carcinoma (p = 0.0004) (Fig 6). Additionally, Kaplan–Meier analysis was conducted based on whether patients with ER-positive BC had received endocrine therapy. The log-rank tests indicated that high *CCBL2* expression was associated with a high RFS rate in patients receiving endocrine therapy (with or without chemotherapy) (p = 0.0039) and in a subgroup of patients receiving endocrine therapy alone (without chemotherapy) (p = 0.0035). Furthermore, high *CCBL2* expression was associated with better OS (p = 0.0020) and RFS (p = 7.2e−05) rate in patients with ER-positive BC without endocrine therapy (Fig 7).

### Independent prognostic value of low *CCBL2* expression in BC

**Fig 8:**
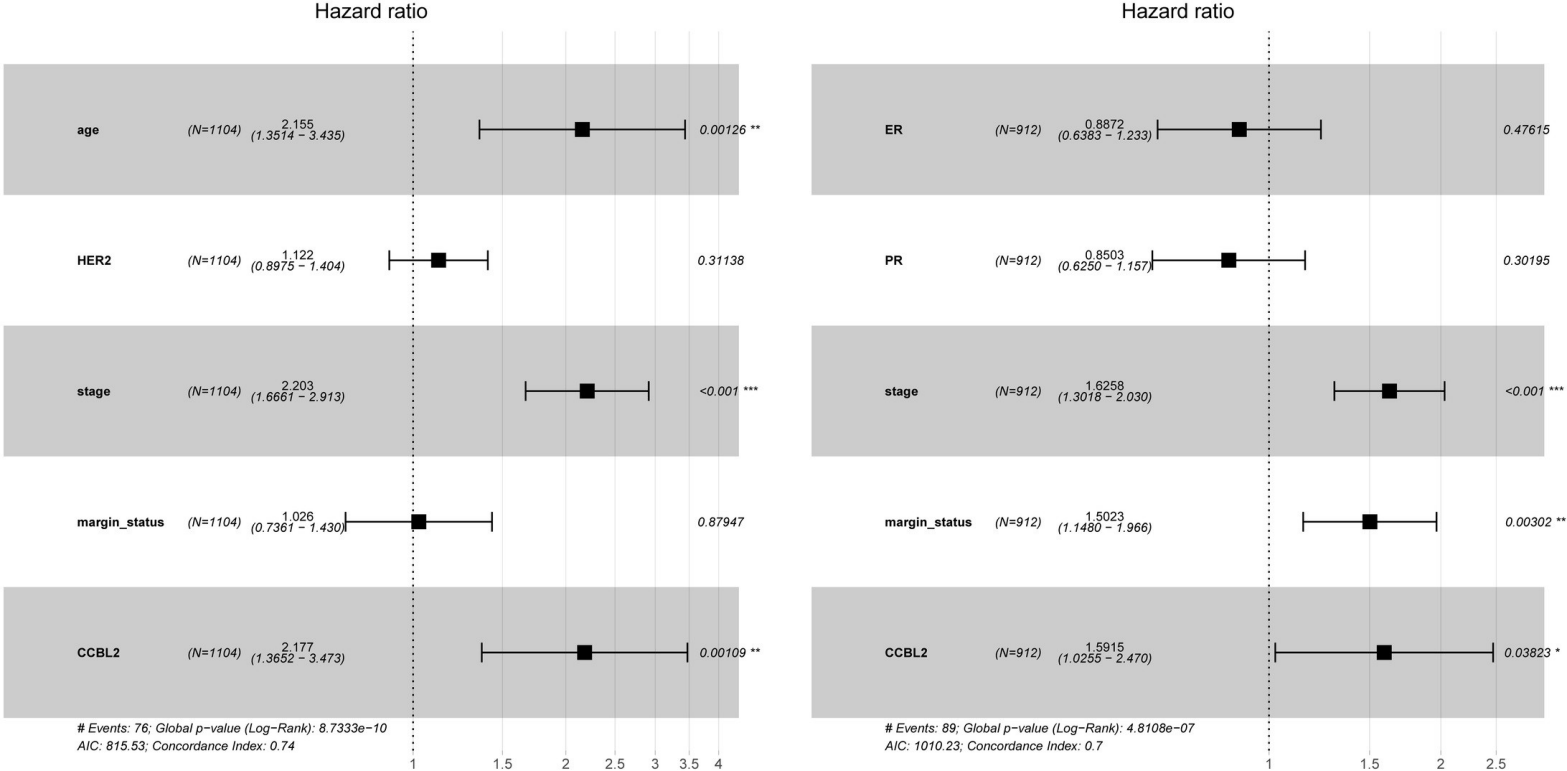
Multivariate analysis was shown in the forest plot.

Univariate and multivariate analyses were performed to demonstrate the prognostic value of clinicopathological characteristics, which were subsequently used in the evaluation of the impacts of *CCBL2* on the survival of patients with BC. Age, clinical stage, HER-2, margin status and *CCBL2* expression were linked with poor OS according to the results of the univariate analysis (Table 3). Likewise, ER, PR, margin status, clinical stage, and *CCBL2* expression were linked with an unfavorable RFS (Table 4). Subsequently, multivariate analysis was performed, the results of which were shown in the forest plot (Fig 8). Low *CCBL2* expression served as an independent prognostic biomarker for low OS (p = 0.0011; HR: 2.18, 95% CI: 1.37–3.47) and low RFS (p = 0.0382; HR: 1.59, 95% CI: 1.03–2.47) (Tables 3 and 4).

**Table 3:** Univariate and multivariate analyses of overall survival in breast cancer patients.

Parameters	Univariate analysis			Multivariate analysis		
	HR	95%CI	P value	HR	95%CI	P value
Age	1.91	1.39–2.63	0	2.16	1.36–3.45	0.0013
Histological type	0.93	0.74–1.17	0.543			
Molecular subtype	1.01	0.88–1.16	0.901			
ER	0.85	0.71–1.02	0.074			
PR	0.87	0.73–1.03	0.096			
HER-2	1.29	1.05–1.57	0.013	1.12	0.90–1.40	0.297
Menopause status	1.16	0.94–1.43	0.165			
Stage	1.64	1.40–1.91	0	2.20	1.67–2.91	0.0000
Margin status	1.42	1.11–1.81	0.005	1.03	0.74–1.43	0.8795
Lymph node status	1.10	0.93–1.30	0.274			
*CCBL2*	2.12	1.53–2.93	0	2.18	1.37–3.47	0.0011

Abbreviations: HR Hazard Ratio, CI confidence interval, bold values of P < 0.05 indicate statistically significant correlations

**Table 4:** Univariate and multivariate analyses of relapse free survival in breast cancer patients.

Parameters	Univariate analysis			Multivariate analysis		
	HR	95%CI	P value	HR	95%CI	P value
Age	1.45	0.97–2.16	0.072			
Histological type	0.86	0.65–1.14	0.290			
Molecular subtype	0.99	0.82–1.2	0.945			
ER	0.78	0.63–0.97	0.026	0.89	0.64–1.23	0.4762
PR	0.78	0.64–0.96	0.019	0.85	0.63–1.16	0.3020
HER-2	0.93	0.7–1.22	0.596			
Menopause status	0.95	0.74–1.22	0.713			
Stage	1.71	1.4–2.08	0	1.63	1.30–2.03	0.0000
Lymph node status	0.86	0.7–1.06	0.159			
Margin status	1.59	1.23–2.06	0	1.50	1.15–1.97	0.0030
*CCBL2*	1.82	1.21–2.73	0.004	1.59	1.03–2.47	0.0382

Abbreviations: HR Hazard Ratio, CI confidence interval, bold values of P < 0.05 indicate statistically significant correlations

### Gene set enrichment analysis (GSEA) of *CCBL2*

**Fig 9:**
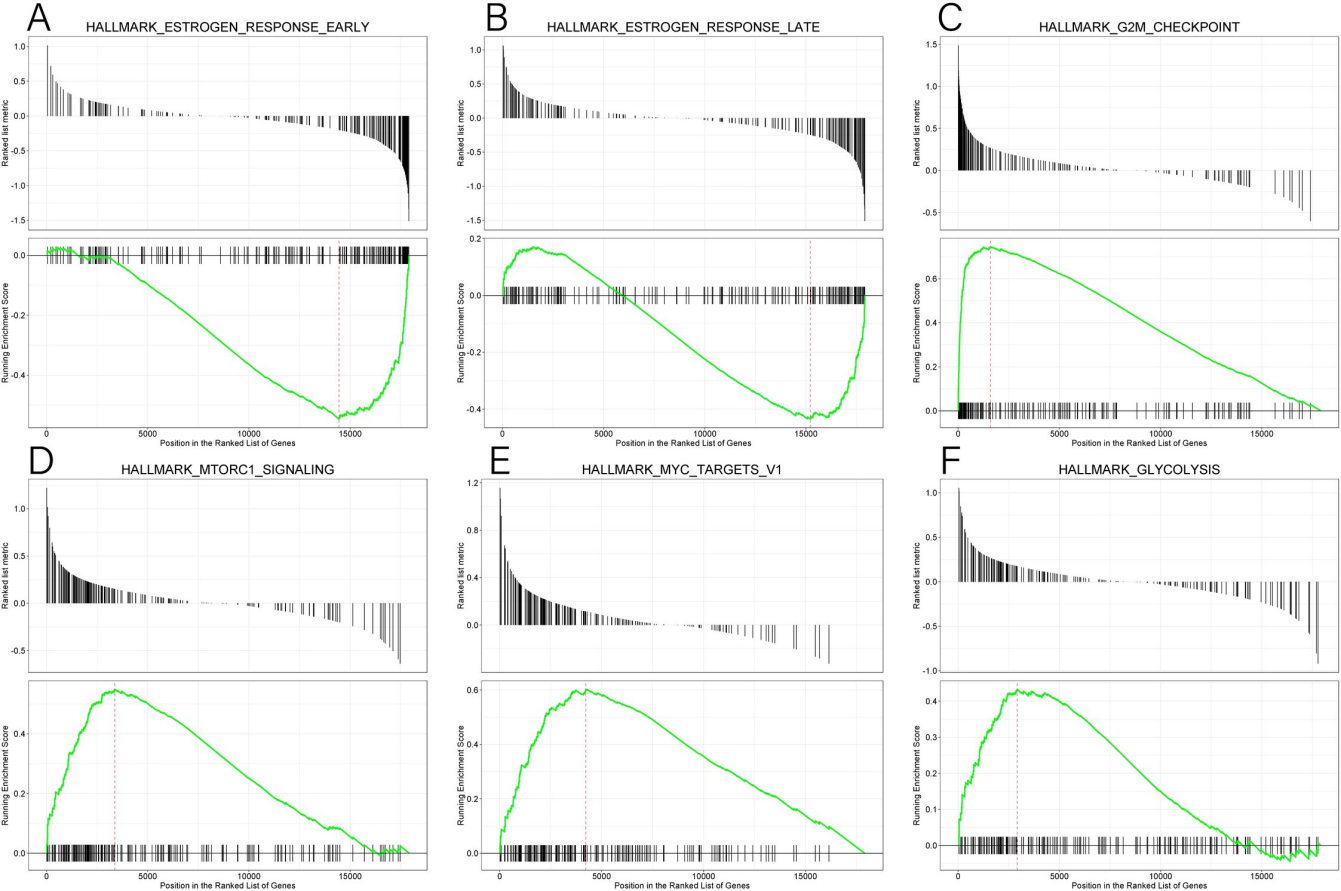
GSEA results of CCBL2 in breast cancer. GSEA results showed the different enrichment of estrogen response early (A), estrogen response late (B), G2M checkpoint (C), mTorc1 signaling (D), MYC targets V1 (E) and glycolysis (F) in *CCBL2* related to breast cancer. Abbreviation: GSEA, Gene Set Enrichment Analysis.

GSEA was performed between the low and high *CCBL2* expression datasets, which was significantly different in h.all.v6.2.symbols.gmt of the MsigDB database (FDR<0.25, NOM p<0.05) (Table 5). On the basis of the normalized enrichment score (NES), the most significantly enriched pathways included estrogen response early and estrogen response late, indicating that the estrogen response was downregulated when *CCBL2* expression was low. In addition, the correlated pathway, androgen response, was also declined (Table 5). The oppositely regulated and enriched pathways included the G2M checkpoint, MYC, mTorc1 signaling, and glycolysis (Fig 9).

**Table 5:** Gene sets which were significantly enriched.

Description of gene set	NES	NOM (p value)	FDR (q value)
**HALLMARK_ESTROGEN_RESPONSE_EARLY**	-2.047	0.001	0.005
**HALLMARK_ESTROGEN_RESPONSE_LATE**	-1.632	0.001	0.005
**HALLMARK_ANDROGEN_RESPONSE**	-1.464	0.015	0.016
**HALLMARK_G2M_CHECKPOINT**	3.164	0.004	0.005
**HALLMARK_MYC_TARGETS_V1**	2.558	0.005	0.005
**HALLMARK_MTORC1_SIGNALING**	2.337	0.005	0.005
**HALLMARK_GLYCOLYSIS**	1.844	0.005	0.005

Abbreviation: NES, normalized enrichment score. FDR, false discovery rate. NOM, nominal p value.

### Correlation of *CCBL2* with other genes in BC

**Fig 10:**
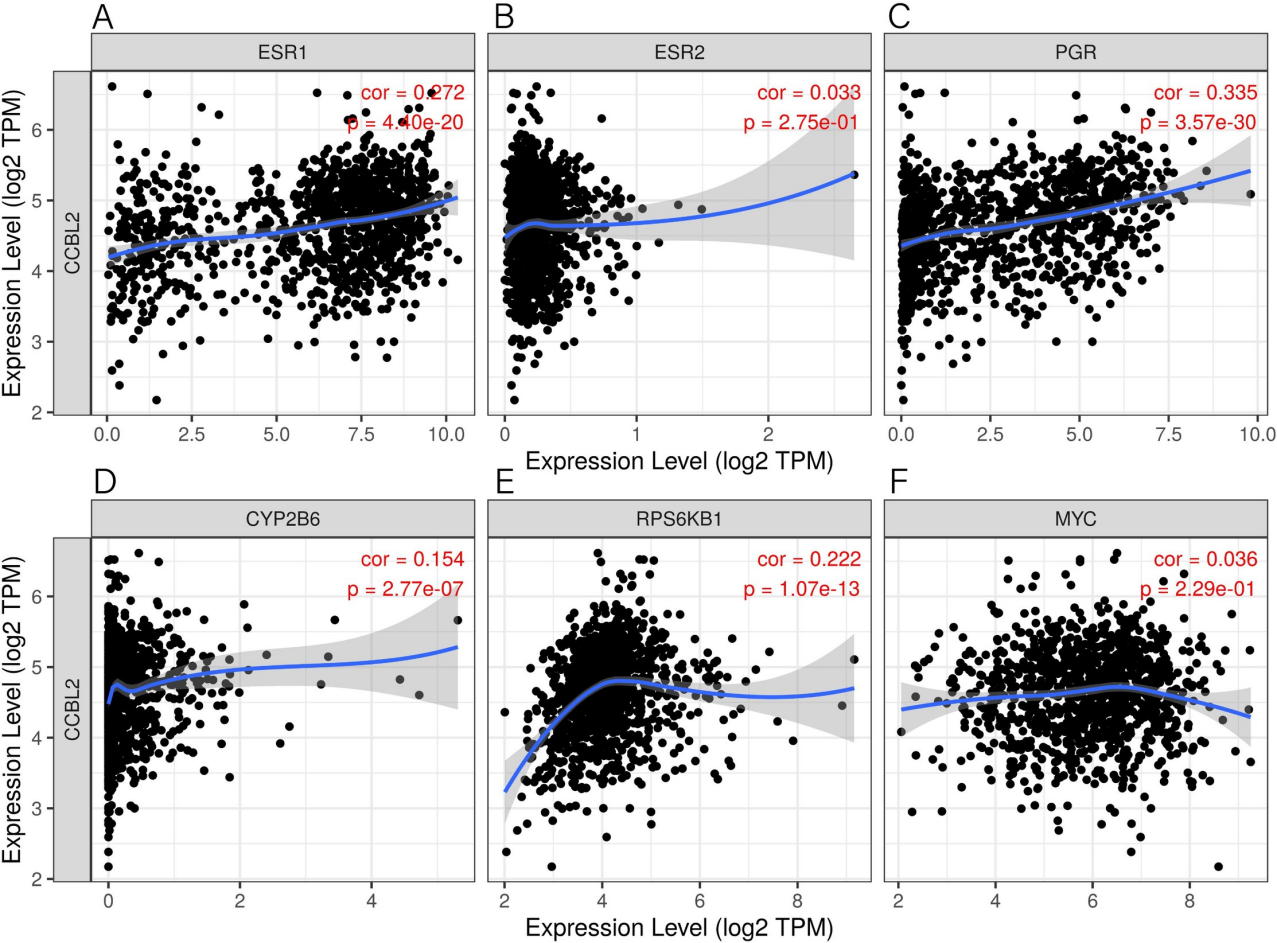
Correlation analysis between *CCBL2* and relevant genes. Results included *ESR1* (A), *ESR2* (B), *PGR* (C), *CYP2B6* (D), *RPS6KB1* (E), and *MYC* (F) through TIMER databases. Abbreviation: TIMER, Tumor Immunoassay Resource.

By the use of TIMER database, we showed that the expression of *CCBL2* was significantly associated with that of *ESR1* (r = 0.2718, p = 4.40e-20), *PGR* (r = 0.3346, p = 3.57e-30), *AR* (r = 0.3412, p = 4.38e-31) (S1 Fig), *CYP2B6* (r = 0.1542, p = 2.77e-07) and *RPS6KB1* (r = 0.2215, p = 1.07e-13), while no significant association with that of *ESR2* (r = 0.0330, p = 2.75e-01) and *MYC* (r = 0.0363, p = 2.29e-01) (Fig 10).

### Pan-cancer analysis of *CCBL2* expression

**Fig 11:**
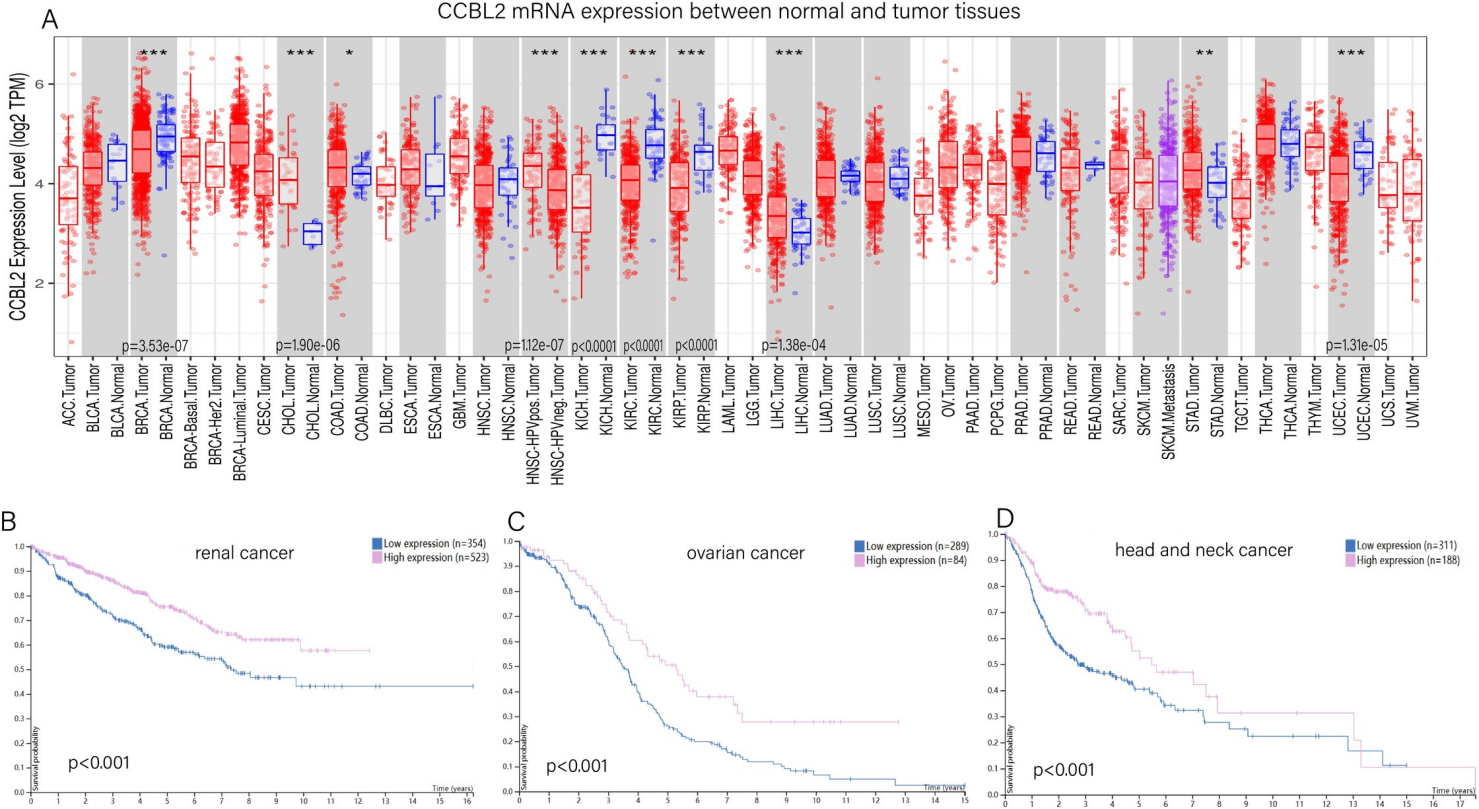
Pan-cancer analysis of *CCBL2*. Differential expression of *CCBL2* between normal and tumor tissues(A). Low *CCBL2* expression had relations with the low survival probability of renal cancer (B) and ovarian cancer (C) and head and neck cancer (D) (p < 0.0010). Data were obtained from Human Protein Atlas Dataset available from proteinatlas.org and TIMER databases.

The differential expression of *CCBL2* between normal and tumor tissues was analyzed using the TIMER database. According to the results, the expression of *CCBL2* was significantly higher in normal tissues compared with that in tumor tissues in not only BC but also in renal cancer (p<0.0001), ovarian cancer (p<0.0001), and uterine corpus endometrial carcinoma (p = 1.31e-05). In cholangiocarcinoma (p = 1.90e-06) and liver hepatocellular carcinoma (p = 1.38e-04), the expression of *CCBL2* was lower in normal tissues compared with tumor tissues. Considering the results of both TIMER and HPA databases, monogenic pan-cancer analysis of *CCBL2* expression (data from HPA) was performed, and the results indicated that apart from BC, low expression of *CCBL2* was associated with poor prognosis of renal, ovarian and head and neck cancers (p<0.0010). There was no significant relation between *CCBL2* and prognosis of patients with uterine corpus endometrial carcinoma (p = 0.1600) and liver cancer (cholangiocarcinoma and liver hepatocellular carcinoma) (p = 0.2600) (Fig 11).

### Low *CCBL2* expression in patient-derived tissue samples of breast, renal, ovarian and head and neck cancers

The results of IHC staining were downloaded from HPA, and IHC staining was employed to verify the protein expression of *CCBL2* in breast, renal, ovarian and head and neck cancers. Compared with the strong IHC staining in normal tissues, there were totally 24 samples of breast cancer. 22 tissue samples exhibited moderate IHC staining and 2 tissue samples exhibited weak staining. Out of 24 renal cancer samples, 3 of them were detected moderate staining, and 21 of them were detected weak staining. For ovarian cancer, 5 tissue samples presented strong staining, 16 showed moderate staining and 5 showed weak staining. A total of 8 tissue samples of head and neck cancer were analyzed, out of which 5 showed moderate staining, and 3 showed weak staining. The first three types of cancer tissues showed evidently weaker staining than their respective normal tissues (para-tumor tissues); however, no change in the staining intensity was observed in tissue samples of head and neck cancer. In particular, in BC, infiltrating lobular carcinoma presented stronger staining than infiltrating ductal carcinoma (Fig 2).

## Discussion

Based on the data acquired from the TCGA database, *CCBL2* showed lower expression in tumor tissues compared with normal tissues, and this result was validated in GEO datasets. In addition, RT-qPCR and IHC staining demonstrated the enhanced expression of *CCBL2* in the human normal breast epithelial cell line MCF-10A and its diminished expression in BC cell lines as well as BC tissues. Low *CCBL2* expression was correlated with an unfavorable survival, and we could come to a conclusion that *CCBL2* was a prognostic biomarker in BC. To the best of our knowledge, this is the first study to elucidate the correlation between *CCBL2* expression and BC survival based on TCGA data analysis.

*CCBL2*, a gene located on chromosome 1p22.2 [11], encodes an aminotransferase that transaminates kynurenine to form kynurenic acid, which is a metabolite of tryptophan. According to previous studies, *CCBL2* facilitated the clearance of nephrotoxic substances [26]. The expression of *CCBL2* was also decreased in patients with hyperoxaluria [27]. Moreover, *CCBL2* expression was positively correlated with the occurrence of hospital-acquired VTE [19]. As important paralogs, evidence has shown the correlation between *CCBL1* (identical to *KAT1* and *GTK*) and pancreatic, prostate, and bladder cancers [28]. Furthermore, *CCBL2* (identical to *KAT2*) plays an important role in several neurological diseases such as Huntington’s disease, Alzheimer’s disease and depression [15, 18, 29]. However, limited information is available regarding the expression of *CCBL2* in tumors, especially BC. In our study, we demonstrated that *CCBL2* expression was lower in tumor tissues than in normal tissues based on both the TCGA database and microarray datasets GSE42568 and GSE71053.

Low expression of *CCBL2* was correlated with several clinicopathologic characteristics, including histological type, ER, PR, HER2, molecular subtype, T classification, M classification, vital status, and stage. Several research groups have reported that the abovementioned clinicopathologic features could guide the diagnosis, treatment and prognosis of BC, which promoted us to further explore the correlation between *CCBL2* and BC [9, 30]. As confirmed above, *CCBL2* had significantly strong relation with ER (p = 0.0005) and PR (p = 0.0005) status, accounting for the lower expression of *CCBL2* in tumor cells of basal-like/Her-2-enriched BC and higher expression in luminal A (ER/PR-positive) BC cells. Therefore, lower OS and RFS were linked with lower *CCBL2* expression because of the low survival rate and poor prognosis of basal-like/Her-2 enriched BC. The prognosis of infiltrating ductal carcinoma was found to be worse than that of infiltrating lobular carcinoma [31]. And the analysis results showed that the expression of *CCBL2* was lower in infiltrating ductal carcinoma than in infiltrating lobular carcinoma, which was consistent with the IHC staining results that lower *CCBL2* expression was linked to worse BC survival. A recent study showed that *CCBL1* (identical to *GTK*) was involved in glutamine utilization through the GLS1 and glutaminase II pathways to generate glutamate [23], while the role of *CCBL2* (identical to *GTL*) was unclarified, although glutamine was one of its metabolic substrates [15]. For this reason, when *CCBL2* expression is low, it can be estimated that glutamine is relatively abundant. Glutamine plays an important role in the biosynthesis of amino acids, proteins, lipids, and nucleotides, which are essential to cell division, especially in cancer cells, also known as glutamine addiction. Therefore, the proliferating BC cells consumed glutamine at a very high rate [32, 33]. Thus, among all T stages, T4 had the lowest *CCBL2* levels and relatively the highest glutamine levels, with the fastest tumor cell growth and angiogenesis. In the case of the M stage, low *CCBL2* levels tended to be related to distant metastasis [23, 34]. Many cancer cells, especially those driven by the *Myc* gene (involving BC, as confirmed by GSEA results), were metabolically reprogrammed to consume more glutamine. When the expression of *CCBL2* was low, the pathway of *Myc* in BC cells was upregulated. Altered glutamine metabolism in *Myc*-driven cancer, BC, resulted in glutamine addiction, which caused worse survival rate [35].

Our study shows that low expression of *CCBL2* is associated with low OS in BC, especially in ER-positive tumors, PR positive tumors, HER-2 negative tumors, luminal A tumors, and invasive ductal and lobular carcinomas. Estrogen plays an important role in BC progression. Through GSEA, we found several relevant pathways, including estrogen response, enriched in *CCBL2*. Estrogen response pathway was downregulated when *CCBL2* exhibited low expression, indicating that *CCBL2* is positively correlated with this pathway. Oshi *et al*. found that the *ESR1*-associated early estrogen response was upregulated in ER-positive BC, indicating a better OS [36]. Therefore, that the low expression of *CCBL2* was correlated with the worse OS of ER-positive BC, which was consistent with the results of our survival analysis. However, the pathway of estrogen response (early) involves 200 relevant genes [36] and the underlying molecular mechanism remains unclear. Additionally, *CCBL2* could favorably predict the response to endocrine therapy in patients with ER-positive BC. Conventionally, RFS is used to evaluate the therapeutic effect of adjuvant therapy in carcinomas. The results of the Kaplan-Meier analysis indicated that with higher *CCBL2* expression, patients who received endocrine therapy showed better RFS rates than lower *CCBL2* groups. More specifically, patients receiving endocrine therapy alone (without chemotherapy) with higher *CCBL2* expression presented a significant better RFS rate, whereas patients receiving both endocrine therapy and chemotherapy showed no increase in OS rate and insignificant increase in RFS rate. Therefore, *CCBL2* possesses a significant prognostic value for ER-positive BC patients with or after endocrine therapy, particularly in the subgroup receiving only endocrine therapy, but little prognostic value in the subgroup receiving both endocrine therapy and chemotherapy. In patients with ER-positive BC without endocrine therapy, high CCBL2 expression indicated a favorable OS and RFS. In other words, CCBL2 also exhibited a valuable prognostic capacity in BC patients without or before endocrine therapy. Nowadays, the administration of endocrine therapy is mainly based on ER status (ER-positive tumors). However, patient compliance is poor due to the requirement for long-term medicine and associated side effects, with 20–50% of patients failing to finish the treatment cycle [37, 38]. Additionally, drug resistance has become a growing problem. Hence, novel biomarkers, such as *CCBL2*, are needed to evaluate the benefit of endocrine therapy, which would allow for a better prognosis. This will not only improve patient compliance but also assist in the selection of patients who are likely to benefit from neoadjuvant endocrine therapy.

Similarly, the mTORC1-signaling pathway is also correlated with ER-positive BC, which was downregulated when the *CCBL2* expression was high. The regulatory targets of rapamycin (mTOR) are involved in protein translation, metabolism, cell growth, and proliferation [39]. As an enzyme complex, MTORC1, mainly binds with S6 kinases (S6Ks) to mediate its function. Studies have demonstrated that *S6K1* (one of the S6 kinases) and some other relevant kinases contributed to the activation of *ERα* through its phosphorylation [40, 41]. It was further shown that *S6K1* and *ERα* formed a positive feed-forward loop. The phosphorylation of *ERα* by *S6K1* facilitated the process, promoting the transcription of *RPS6KB1*, which in turn regulated he proliferation of BC cells [42, 43]. To conclude, lower expression of *CCBL2* in ER-positive or luminal A BC is related to the higher transcription of *RPS6KB1* and subsequent BC cell proliferation. For the reasons mentioned above, the consensus can be reached that lower *CCBL2* expression is associated with worse OS in ER-positive or luminal A BC. Moreover, the *Myc* pathway is negatively correlated with *CCBL2* according to the GSEA. In triple-negative breast cancer (TNBC), MYC was found to regulate polyamine metabolism and a plasma polyamine signature was related to the development and progression of TNBC [44]. Therefore, for TNBC, the lower expression of *CCBL2* was related to worse OS and RSF, consistent with the Kaplan–Meier survival analysis. However, to date, not much is known about the correlation between *CCBL2* and *Myc*, thereby warranting further experimental validation. Other results of GSEA stated that G2M-checkpoint and glycolysis pathways were negatively correlated with *CCBL2* expression, both of which have close correlation with the proliferation of most types of malignant tumors [45]. This is obviously consistent with the results of our analysis.

In addition to ER and PR, AR is also a hormone receptor that is expressed on the surface of mammary cells, regarded as a novel biomarker arousing heated discussion. Our study showed that the expression of *CCBL2* was positively correlated with that of the AR gene and that the pathway of androgen response was upregulated when *CCBL2* expression was high. According to a prospective clinical study by Anand et al., higher AR expression was correlated with earlier stage (p < 0.03), lower axillary burden (p < 0.04), higher ER (p = 0.002) and PR (p = 0.001) expression [46]. More specifically, Lin et al. showed that AR expression was positively correlated with a better prognosis of patients with HER2-positive breast carcinomas [47]. In TNBC, several studies have confirmed that the luminal androgen receptor (LAR) subtype, defined as AR-positive subtype, was associated with the highest OS compared with other subtypes [48–50]. We can conclude that *CCBL2* could be a potential prognostic biomarker based on the confirmed relationship between higher AR and better BC prognosis, as well as the findings of the study mentioned above.

Furthermore, we found some proofs to confirm our conclusions with the assistance of website of http://guotosky.vip:13838/GPSA/. In this website, 3048 gene knock out RNAseq datasets were performed GSEA with four source of gene sets, including TCGA, Genotype-Tissue Expression (GTEx), and Cancer Cell Line Encyclopedia(CCLE). The result (S1 Table) showed the negative fold change values of *ESR1* and *AR* with knock-down *CCBL2* gene. It could be assumed that when *CCBL2* gene was knocked down, *ESR1* and *AR* genes also declined.

Using multivariate analysis, it was confirmed that low *CCBL2* expression might serve as an independent prognostic marker, which was correlated with the unfavorable OS and RFS of BC using multivariate analysis. Then, by analyzing the AUC value, we found that *CCBL2* possessed a moderate diagnostic efficacy between tumor and normal tissues, especially in stage IV. Therefore, *CCBL2* can be regarded as a novel biomarker in the field of diagnosis and prognosis in BC.

The pan-cancer analysis revealed differential expression of *CCBL2* between normal human tissues and various types of cancers. In addition to BC, some other cancers exhibited significantly different *CCBL2* expression levels. Combined with the HPA results, renal, ovarian and head and neck cancers had significantly different *CCBL2*-related survival rates. According to the findings of RT-qPCR and IHC, *CCBL2* may be a favorable prognostic biomarker in most cancer types. Further experimental verification was underway. Further *in silico* and *in vitro* research is needed to explore the correlation between *CCBL2* and pan-cancers.

In this study, we initially discussed the value of *CCBL2* expression as an independent prognostic marker for BC. Due to the limited samples, further explorations still need to be carried out based on the large samples data to verify our consequence. Additionally, the results of this study also promote the subsequent work, involving the further cell function test through gene overexpression or knock-down.

## Conclusion

Compared with normal tissues, the expression of *CCBL2* was lower in BC. The results revealed that low *CCBL2* expression could act as an independent prognostic marker associated with survival of BC.

## Supporting information

S1 Fig Correlation analysis between *CCBL2* and AR through TIMER databases. Abbreviation: AR, Androgen Receptor; TIMER, Tumor Immunoassay Resource.
(TIF)

S1 Table The predictive results of *CCBL2* knock-down. Abbreviations: LogFC Log2 fold-change, AveExpr average log2-expression, P value< 0.05 indicate statistically significant correlations.
(DOCX)

S1 File CCBL2 raw data. (XLSX)

S2 File PCR results. (ZIP)

S3 File GSE42568 data. (ZIP)

S4 File GSE71053 data. (ZIP)

## List of abbreviations

AR: Androgen receptor
AUC: Area under the curve
BC: Breast cancer
CCBL2: Cysteine conjugate beta-lyase 2
ER: Estrogen receptor
GEO: Gene Expression Omnibus
GSEA: Gene Set Enrichment Analysis
GTK: Glutamine Transaminase K
GTL: Glutamine Transaminase L
HER-2: human epidermal growth factor-2
HPA: The human protein atlas
HR: Hazard ratio
IHC: Immunohistochemistry
KAT3: Kynurenine aminotransferase 3
OS: Overall survival
PR: progesterone receptor
RFS: Relapse-free survival
ROC: Receiver operating characteristic
RT-qPCR: Real-time quantitative polymerase chain reaction
TCGA: The Cancer Genome Atlas
TIMER: Tumor Immunoassay Resource
