# Supplementary figures and images for: Cysteine conjugate beta-lyase 2 (CCBL2) expression as a prognostic marker of survival in breast cancer patients
Source: PLoS One. 2022 Jun 30;17(6):e0269998. doi: 10.1371/journal.pone.0269998 (PMC9246202; doi:10.1371/journal.pone.0269998)

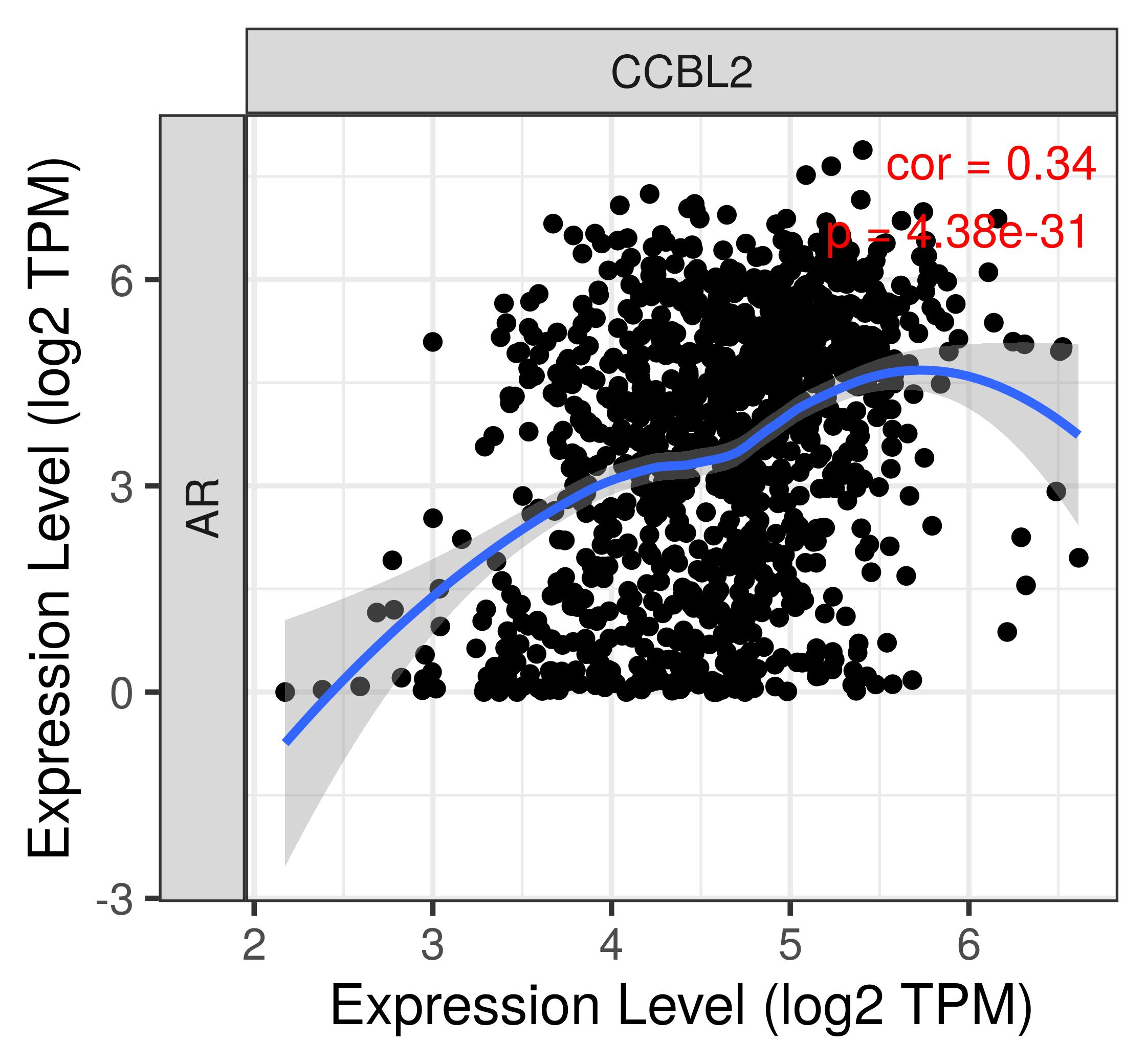

Supplement: S1 Fig — Abbreviation: AR, Androgen Receptor; TIMER, Tumor Immunoassay Resource. (TIF) [file pone.0269998.s001.tif]
