# Supplementary material for: Cysteine conjugate beta-lyase 2 (CCBL2) expression as a prognostic marker of survival in breast cancer patients
Source: PLoS One. 2022 Jun 30;17(6):e0269998. doi: 10.1371/journal.pone.0269998 (PMC9246202; doi:10.1371/journal.pone.0269998)
Supplement: S1 Table — Abbreviations: LogFC Log2 fold-change, AveExpr average log2-expression, P value< 0.05 indicate statistically significant correlations. (DOCX) [file pone.0269998.s002.docx]

**S1 Table.** **The predictive results of *CCBL2* knock-down**

| **Knock-down gene** | **Gene** | **LogFC** | **AveExpr** | **P.Value** | **Adj.P.Val** | **Celline** | **Method** |
| --- | --- | --- | --- | --- | --- | --- | --- |
| CCBL2 | ESR1 | -0.2479 | 979.3983 | 0.0038 | 0.0135 | MCF7 | siRNA |
| CCBL2 | AR | -0.7272 | 184.2677 | 6.44E-07 | 6.40E-06 | 22Rv1 | siRNA |

Abbreviations: LogFC Log2 fold-change, AveExpr average log2-expression, P value< 0.05 indicate statistically significant correlations
